# Molecular structure and nickel-binding capacity of *Proteus mirabilis* UreE

**DOI:** 10.1107/S2059798326001907

**Published:** 2026-03-16

**Authors:** Jiayi Pan, Sarah L. Mueller, Nuren Tasneem, Yang Wu, Emily J. Furlong

**Affiliations:** a Division of Biomedical Science and Biochemistry, Research School of Biology Australian National University Canberra ACT Australia; b Division of Plant Sciences, Research School of Biology Australian National University Canberra ACT Australia; c Research School of Earth Sciences Australian National University Canberra ACT Australia; McGill University, Canada

**Keywords:** metalloproteins, nickel chaperones, *Proteus mirabilis*

## Abstract

UreE is a nickel chaperone that is required for the safe and efficient delivery of nickel to the active site of the metalloenzyme urease, which is a key virulence factor of the urinary-tract pathogen *Proteus mirabilis*. We investigated the structural features of *P. mirabilis* UreE (*Pm*UreE) using protein X-ray crystallography and its nickel-binding capacity by inductively coupled plasma mass spectrometry. Here, we report a 2.0 Å resolution crystal structure of homodimeric *Pm*UreE and show that it has the capacity to bind five Ni(II) ions per dimer. Truncation of the histidine-rich C-terminus reduced the nickel-binding capacity by two Ni(II) ions per dimer, and comparison with homologous UreE structures allowed the assignment of putative nickel-binding sites within the *Pm*UreE structure. These findings increase our understanding of how *Pm*UreE binds nickel and ultimately prevents this toxic metal from causing significant cellular damage in *P. mirabilis*.

## Abstract 2

This study presents a 2.0 Å resolution crystal structure and determined the nickel-binding capacity of the *P. mirabilis* nickel chaperone UreE. UreE is essential for the delivery of nickel to the metalloenzyme urease, which is key for the ability of *P. mirabilis* to cause urinary-tract infections.

## 1. Introduction

*Proteus mirabilis* is a Gram-negative bacterium and a leading contributor to catheter-associated urinary-tract infections (CAUTIs), known for its ability to induce crystalline biofilms and the formation of urinary stones (Armbruster *et al.*, 2017 ▸, 2018 ▸). Crystalline biofilms and urinary stones block catheters but also protect *P. mirabilis* from the immune system and antibiotics, enabling persistent infection of the urinary tract (Stickler & Feneley, 2010 ▸; Sabbuba *et al.*, 2004 ▸). The critical protein responsible for the crystal-forming trait of *P. mirabilis* is urease (Jones *et al.*, 1990 ▸; Mobley & Hausinger, 1989 ▸). Urease is a nickel-dependent enzyme that hydrolyses urea and produces ammonia, which subsequently increases the pH of the local environment. In the urinary tract, this causes the formation of struvite and apatite crystals from the minerals found in urine (Schaffer *et al.*, 2016 ▸; Li *et al.*, 2002 ▸). Active urease is required for *P. mirabilis* pathogenesis; mutants that do not express the enzyme or are deficient in nickel import cannot induce urinary-stone formation and have significantly reduced fitness in a murine urinary-tract infection (UTI) model (Jones *et al.*, 1990 ▸; Brauer *et al.*, 2020 ▸).

Despite its necessity for urease activity, nickel can also be toxic to bacteria at high intracellular concentrations, disrupting cellular homeostasis and inducing oxidative stress. Therefore, bacterial nickel uptake and distribution must be strictly controlled to prevent toxicity and ensure appropriate metal allocation to nickel-dependent enzymes (Zeer-Wanklyn & Zamble, 2017 ▸). Most research on the maturation processes of bacterial ureases has been performed in *Klebsiella aerogenes* and *Helicobacter pylori*. In these organisms, the nickel chaperone UreE has an essential role in capturing nickel in the bacterial cytoplasm and delivering it to UreG, which then goes on to interact with UreD and UreF to deliver the toxic metal into the urease active site (Nim *et al.*, 2023 ▸; Nim & Wong, 2019 ▸).

Structural and biochemical analyses of UreE from *H. pylori*, *K. aerogenes* and *Sporosarcina pasteurii* have shown that it functions as a dimer (Remaut *et al.*, 2001 ▸; Shi *et al.*, 2010 ▸; Song *et al.*, 2001 ▸; Banaszak *et al.*, 2012 ▸; Zambelli *et al.*, 2013 ▸). The core fold is conserved between these species and includes a GNRH motif that sits at the dimer interface, with the histidine residues from each protomer contributing to the coordination of one Ni(II) ion. There are variations between UreE homologues, primarily in the C-terminus, which often features a histidine-rich tail with variable length and amino-acid composition (Supplementary Fig. S1). As a result of this, the number of nickel ions bound by UreE homologues and the nickel-binding sites also vary. *H. pylori* only has one histidine in its C-terminal region and studies report an overall nickel capacity of approximately one Ni(II) ion per dimer, which is coordinated between the histidine residues at the dimer interface and one C-terminal histidine (Bellucci *et al.*, 2009 ▸; Shi *et al.*, 2010 ▸; Banaszak *et al.*, 2012 ▸). *S. pasteurii* (*Sp*) has two C-terminal histidine residues, and isothermal titration calorimetric and crystallographic results showed two Ni(II) ions binding per dimer with positive cooperativity between the two sites (Stola *et al.*, 2006 ▸). The high-affinity site involves the conserved dimer-interface site and one C-terminal *Sp*His145, while the second site involves *Sp*His145 and *Sp*His147 from the other protomer (Stola *et al.*, 2006 ▸; Zambelli *et al.*, 2013 ▸). *K. aerogenes* (*Kp*) UreE has ten C-terminal histidine residues and binds approximately six Ni(II) ions per dimer, whereas a C-terminally truncated variant (*Ka*H144*UreE, lacking the His-rich tail) binds only two Ni(II) ions per dimer (Lee *et al.*, 1993 ▸; Brayman & Hausinger, 1996 ▸). The crystal structure of *Ka*H144*UreE, however, supports the binding of three metal ions, suggesting that the earlier experiments may underestimate the nickel-binding capacity (Song *et al.*, 2001 ▸). The *Ka*H144*UreE variant remains functional for urease activation, indicating that the core dimer can support the delivery of Ni(II) ions to urease via UreG without the C-terminal histidine residues, albeit with slightly less efficiency (Brayman & Hausinger, 1996 ▸). Further mutational studies in *Ka*UreE identified another nickel-binding site within the core fold of each UreE protomer composed of two histidine residues (*Ka*His110 and *Ka*His112), which is not conserved in *Hp*UreE or *Sp*UreE (Colpas *et al.*, 1999 ▸; Song *et al.*, 2001 ▸).

Given the important role that UreE has in urease activation and subsequent pathogenicity, we sought to structurally characterize *P. mirabilis* UreE (*Pm*UreE) and investigate its metal-binding capacity. Here, we present a 2.0 Å resolution crystal structure of *Pm*UreE and show that it can bind five Ni(II) ions per dimer, with the histidine-rich C-terminal tails contributing to the coordination of two Ni(II) ions per dimer. This work increases our knowledge about the diversity of UreE homologues and provides insight into how UreE binds nickel within *P. mirabilis*.

## 2. Materials and methods

### 2.1. Sequence alignment

A multiple-sequence alignment of UreE from *P. mirabilis* (UniProt ID P17090), *K. aerogenes* (UniProt ID P18317), *H. pylori* (UniProt ID B6JPH3) and *S. pasteurii* (UniProt ID P50049) was performed using *ClustalW* (Higgins *et al.*, 1996 ▸) and visualized with *ESPript*3 (Gouet *et al.*, 1999 ▸).

### 2.2. Molecular cloning and mutagenesis

To generate the *Pm*UreE expression plasmid (pETHis6TEVLIC_UreE), the *P. mirabilis* ATCC 12453 *ureE* open reading frame (486 bp) and the kanamycin-resistant pETHis6TEVLIC plasmid backbone were amplified separately by PCR using Q5 High-Fidelity 2× Master Mix (New England Biolabs; primers are listed in Supplementary Table S1). The pETHis6TEVLIC cloning vector (1B) was a gift from Scott Gradia (Addgene plasmid No. 29653; https://n2t.net/addgene:29653; RRID Addgene_29653). Reaction setup and reagent volumes followed the vendor’s instructions. In the amplification of the backbone, the region encoding the N-terminal His_6_-tag and TEV protease cleavage site was removed. The insert and backbone were assembled using NEBuilder HiFi DNA Assembly Master Mix. Chemically competent *Escherichia coli* Top10 cells were transformed with the assembly. Transformants were selected on kanamycin plates and colonies were screened by PCR with NEBuilder Quick-Load Taq 2× Master Mix.

To generate the *Pm*UreE-ΔC17 truncation mutant expression plasmid, a stop codon was inserted into the pETHis6TEVLIC_UreE construct at the intended truncation site (after Gly144), utilizing the New England Biolabs site-directed mutagenesis protocol, with the Q5 High-Fidelity 2× Master Mix (primers in Supplementary Table S1) and KLD Enzyme Mix. The resulting product was transformed into chemically competent NEB *E. coli* Turbo cells and plated on kanamycin plates.

For both expression constructs, plasmid DNA of positive clones was isolated using the NEB Monarch Spin Plasmid Miniprep Kit. The plasmid sequences were confirmed by whole-plasmid Nanopore sequencing (Plasmidsaurus; Supplementary Fig. S2).

### 2.3. Protein expression and purification

*E. coli* BL21(DE3) pLysS transformed with the pETHis6TEVLIC_UreE plasmid or *E. coli* BL21 (DE3) carrying pETHis6TEVLIC_UreE-ΔC17 was cultured overnight in LB medium supplemented with 50 µg ml^−1^ kanamycin and 34 µg ml^−1^ chloramphenicol (for the cells with the pLysS plasmid). The starter culture was diluted 1:100 into 1 l Terrific Broth (TB) containing the same antibiotic concentrations. The cultures were incubated at 310 K and 180 rev min^−1^ until the optical density at 600 nm (OD_600_) reached 0.8–1.0. Protein expression was induced with 1 m*M* isopropyl β-d-1-thio­galactopyranoside (IPTG), followed by further incubation at 310 K for 4 h. The cells were harvested by centrifugation at 4500 rev min^−1^ (Beckman Coulter Avanti JE, JLA-9.1000 Rotor) for 15 min at 277 K. Both proteins were expressed without affinity tags and were purified following a similar method to that reported previously (Sriwanthana *et al.*, 1994 ▸).

All purification steps were performed at 277 K unless otherwise specified. Cell pellets were resuspended in buffer *A* (20 m*M* Tris–HCl pH 7.5, 500 m*M* NaCl) supplemented with a protease-inhibitor tablet (SIGMAFAST Protease Inhibitor Tablets, Sigma–Aldrich) and DNase I, and lysed using a Qsonica Q700 probe sonicator (cycles of 10 s on and 20 s off for a total of 5 min at 40% amplitude). The lysate was clarified by centrifugation (30 000*g*, 30 min) and the supernatant was loaded onto 2 or 4 ml Ni–NTA His-Bind Resin (EMD Millipore; catalogue No. 70666-5) pre-equilibrated in buffer *A* and packed in a gravity-flow column. After binding, the column was washed with buffer *B* (buffer *A* + 20 or 60 m*M* imidazole) and bound protein was eluted with buffer *C* (buffer *A* + 250 m*M* imidazole). Eluted fractions were pooled and concentrated in a 3 kDa molecular-weight cutoff (MWCO) centrifugal concentrator, filtered (0.22 µm) and subjected to size-exclusion chromatography on a HiLoad 16/600 Superdex 75 column (ÄKTApure system, Cytiva) pre-equilibrated in SEC buffer (20 m*M* HEPES pH 7.5, 100 m*M* NaCl with or without 1 m*M* EDTA). Elution was monitored at 280 nm, and peak fractions were analysed by SDS–PAGE on Bolt 4–12% Bis-Tris Plus Protein Gels in MES buffer (Thermo Fisher Scientific). Fractions corresponding to dimeric *Pm*UreE/*Pm*UreE-ΔC17 were collected. The purified protein was concentrated in 3 kDa MWCO concentrators and then aliquoted, flash-frozen in liquid nitrogen and stored at 193 K. The total yield of *Pm*UreE was ∼109 mg from 4.5 g of cell pellet and that of *Pm*UreE-ΔC17 was ∼41 mg from 3.4 g of cell pellet.

*Pm*UreE and *Pm*UreE-ΔC17 concentrations were determined by measuring the absorbance at 280 nm using a QuickDrop spectrophotometer (SpectraMax) and dividing the measurement by the *ProtParam*-generated theoretical extinction coefficient of 0.736 or 0.728, respectively.

### 2.4. Crystallization

The *Pm*UreE protein used for crystallization was purified without the addition of EDTA. Initial crystallization trials were performed in vapour-diffusion sitting drops on 96-well MRC 2-well plates using a Formulatrix NT8 microfluidics protein crystallization robot. Four commercial screens were tested across protein concentrations ranging from 3 to 20 mg ml^−1^: Shotgun (Molecular Dimensions; Abrahams & Newman, 2021 ▸), JCSG-*plus* (Molecular Dimensions), PEGRx (Hampton Research) and Crystal Screen HT (Hampton Research). Drops were set up with 100 nl protein solution and 100 nl reservoir solution (1:1), equilibrated against 50 µl reservoir solution and incubated at 293 K. Needle-shaped crystals were obtained in 0.2 *M* ammonium citrate dibasic, 20%(*w*/*v*) polyethylene glycol (PEG) 3350 (JCSG-*plus* condition A3) with 5 mg ml^−1^*Pm*UreE within 5–7 days. No other crystallization hits were obtained in the screens. Optimization trials around the initial hit were conducted using 24-well VDX plates with sealant (Hampton Research) by hanging-drop vapour diffusion at 293 K with drops containing 1 µl protein solution and 1 or 2 µl reservoir solution equilibrated against 500 µl reservoir solution. As systematic variation of PEG percentage and salt concentration and additives produced only modest improvements, micro-seeding was performed as reported previously (D’Arcy *et al.*, 2014 ▸; Bergfors, 2007 ▸). The seed stock was prepared from needle-like crystals grown in 0.14–0.22 *M* ammonium citrate dibasic, 16–22%(*w*/*v*) PEG 3350 by crushing the crystals in a small volume of mother liquor; this was then serially diluted 100-fold, 1000-fold and 10 000-fold.

Robot-assisted re-screening was then performed across the same four screens (Shotgun, JCSG-*plus*, PEGRx and Crystal Screen) at a protein concentration of 5 mg ml^−1^ using a sitting-drop setup of 100 nl protein solution, 100 nl reservoir solution and 50 nl seed stock at 293 K. Both undiluted seed stock and a 100-fold dilution were tested. Many more hits were obtained and those conditions that produced well formed crystals were then transferred to a 24-well hanging-drop optimization tray with seeding, using 1 µl protein solution, 1 µl reservoir solution and 0.50 µl seed stock or 1 µl protein solution, 1 µl reservoir solution and 0.25 µl seed stock. During 24-well plate optimization, a 100-fold seed dilution caused over-nucleation (dense microcrystals), so 1000-fold and 10 000-fold dilutions were used for subsequent drops. During optimization, 5% glycerol was added as an additive in some trials to stabilize the crystals. In parallel, the published crystallization conditions reported for UreE homologues were also tested as additional leads (Shi *et al.*, 2010 ▸). Large rod-shaped crystals were obtained after 1–3 days in 0.1 *M* sodium citrate pH 5.0–5.5, 13–19%(*w*/*v*) PEG 8000 or PEG 6000 with or without 5%(*w*/*v*) glycerol and were selected for X-ray diffraction analysis. Despite the morphological improvements, the crystals were fragile, so to limit handling they were cryoprotected by adding 2 µl 40%(*v*/*v*) glycerol directly to the crystallization drop. Crystals were mounted and immediately flash-cooled in liquid nitrogen before being stored at cryogenic temperatures. The best diffracting (1.86 Å resolution) crystal was grown in 0.1 *M* sodium citrate pH 5, 15% PEG 6000, 5% glycerol.

### 2.5. X-ray data collection, structure solution and refinement

X-ray diffraction data were collected on the MX2 microfocus macromolecular crystallography beamline at the Australian Synchrotron, Melbourne, Victoria, Australia (McPhillips *et al.*, 2002 ▸) through remote access using the HTML5 Virtual Network Computing (VNC) client Guacamole. 3600 frames of 0.1° rotation with a total exposure time of 36 s were collected using an Dectris EIGER 16M detector at a wavelength of 0.9537 Å. Raw data were automatically processed at the beamline using *XDS* (*X-ray Detector Software*; Kabsch, 2010 ▸) in space group *P*2_1_2_1_2_1_ and used as input for re-scaling in *AIMLESS* (Evans & Murshudov, 2013 ▸), as implemented in the *Collaborative Computational Project No*. 4 (*CCP*4) suite (version 2.8.5; Agirre *et al.*, 2023 ▸). The high-resolution cutoff was set to 2.0 Å to improve the *I*/σ(*I*) and *R* statistics. The expected solvent content and the likely number of macromolecular copies per asymmetric unit were estimated with the Matthews coefficient tool in *CCP*4 (Matthews, 1968 ▸, Kantardjieff & Rupp, 2003 ▸).

Molecular replacement (MR) was carried out with *Phaser-MR* (maximum-likelihood MR; McCoy *et al.*, 2007 ▸) in the *Phenix* (*Python-based Hierarchical ENvironment for Integrated Xtallography*) suite (version 1.21.2-5419; Liebschner *et al.*, 2019 ▸; Adams *et al.*, 2010 ▸). The search model was the *AlphaFold* prediction of *Pm*UreE from the UniProt database (AF-P17090-F1, UniProt ID P17090; Jumper *et al.*, 2021 ▸). The model was prepared in *phenix.process_predicted_model* to remove low-confidence regions and replace values in the *B*-factor field. Candidate copy numbers suggested by the Matthews analysis were tested and the solution was selected based on translation-function *Z*-score (TFZ) and log-likelihood gain (LLG).

Initial model refinement was performed in *phenix.refine* (Afonine *et al.*, 2012 ▸). Manual model building based on the refined structure was performed in *Coot* (*Crystallographic Object-Oriented Toolkit*; version 0.9.8.95; Emsley *et al.*, 2010 ▸). Side-chain conformations, backbone geometry and alternate conformations were adjusted against the electron-density maps. Each manual editing cycle was followed by an additional round of refinement in *Phenix* until no significant features remained in the difference maps and the model statistics converged. Further refinement included adding water molecules where the difference in density and hydrogen-bonding geometry supported placement. Model validation was performed using *MolProbity* (Williams *et al.*, 2018 ▸; as implemented in *Phenix*) to assess Ramachandran statistics, rotamers, clashscore and overall geometry. Data-to-model agreement was monitored throughout using *R*_work_/*R*_free_. Coordinates and structure factors were deposited in the Protein Data Bank as entry 9zlo and crystallographic statistics are shown in Table 1 ▸. Structural alignments were performed in *Coot* using the ‘Secondary Structure Matching (SSM) Superpose’ command for homologous structures and ‘Least Squares (LSQ) Superpose’ for *Pm*UreE chains and the *AlphaFold* model (Emsley *et al.*, 2010 ▸). Figures were made using *PyMOL* (version 3.1.6.1; Schrödinger).

### 2.6. Inductively coupled plasma mass spectrometry (ICP-MS) quantification of nickel binding

Quantification of the Ni(II) ions associated with *Pm*UreE and *Pm*UreE-ΔC17 was performed using ICP-MS. Samples (typically 20 µ*M* protein in 500 µl incubation buffer) were incubated with 100 µ*M* NiCl_2_ at room temperature for 2.5 h and then dialysed overnight at 277 K against 1 l dialysis buffer to remove free metal ions. The protein concentration was measured post-dialysis for subsequent stoichiometry calculations. For digestion, ∼10 µ*M* protein was mixed in a 1:1 ratio with 4%(*v*/*v*) nitric acid and incubated overnight at room temperature to release bound metal. Process blanks (dialysis buffer and 4% HNO_3_) were prepared in parallel to test the background metal content. Digests were centrifuged (20 000*g*, 10 min, room temperature) and supernatants were collected for analysis.

The analysis was performed on a Thermo Scientific iCAP RQ ICP-MS system in KEDS (kinetic energy dispersion sensitive) mode using helium as a collision gas. A multi-element standard was used for ^60^Ni calibration (Agilent IntelliQuant 68 multi-element standard, Part No. 5190-9422). A five-point external calibration was prepared for ^60^Ni at concentrations of 1, 10, 50, 100 and 500 p.p.b. (µg l^−1^) in the same acid matrix as the samples (2% HNO_3_), yielding a linear regression coefficient (*R*^2^) of ≥0.999. The software *Qtegra* was used to process the data. A mid-range quality-control (QC) standard was prepared independently from the calibration standards and measured before and after the sample sequence to assess and correct for instrumental drift during the run time. Instrumental drift correction was performed using linear interpolation, where the rate of drift was calculated from the rate of change in 100 p.p.b. QC values obtained at the beginning and the end of the run queue and was applied to each sample as a function of its position in the queue. Each sample was analysed in triplicate and the average was used for calculations. Limit of detection (LOD) and limit of quantification (LOQ) were defined from measured blanks (in p.p.b.) as LOD = 3 × standard deviation (SD)_blank, LOQ = 10 × SD_blank (SD_blank from ≥3 independent blank digests). Measurements below LOD were flagged as below detection limit (BDL) and excluded from stoichiometry calculations.

Instrument output (µg l^−1^, p.p.b.) was corrected for the total dilution factors to obtain the ^60^Ni concentration in the post-dialysis sample. Where required, concentrations were converted to micromolar using the molar mass of ^60^Ni, and nickel:protein ratios were calculated using the measured protein concentration and the oligomeric state specified for the experiment (see the formulae below). Results are reported as mean ± SD of three independent experiments.
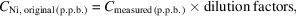

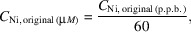

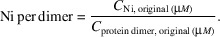


## 3. Results and discussion

### 3.1. Crystallization and structure analysis of *Pm*UreE 

**Figure 1:**
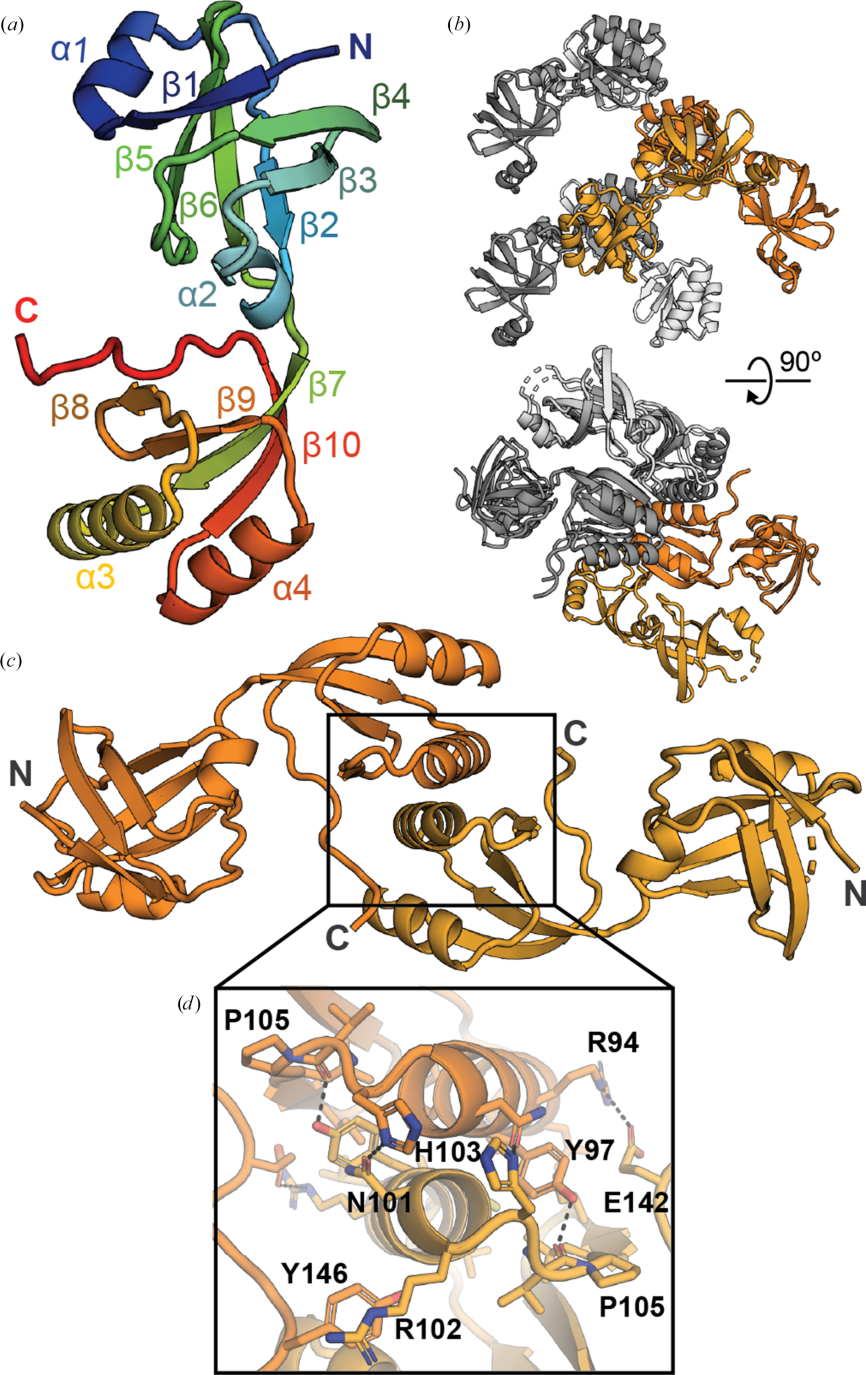
Crystal structure of *Pm*UreE. (*a*) Cartoon representation of *Pm*UreE with secondary-structure features labelled and coloured in rainbow, with the N-terminal region shown in blue/green and the C-terminal region shown in orange/red. (*b*) Crystal packing of the *Pm*UreE crystal structure. The asymmetric unit is coloured in orange (chain *A* is darker orange; chain *B* is lighter orange), while symmetry mates are coloured in grey (chain *A* equivalents are dark grey; chain *B* equivalents are light grey). The biologically relevant dimer is formed through crystallographic symmetry. (*c*) Cartoon representation of the *Pm*UreE dimer with chain *A* coloured dark orange and chain *B* coloured light orange. (*d*) Closer view of the dimerization interface, with key residues labelled and selected hydrogen-bonding interactions represented with dashed black lines.

The full-length *Pm*UreE protein was purified by immobilized metal-affinity chromatography using its native ability to bind nickel, followed by size-exclusion chromatography (SEC; Supplementary Fig. S3). The elution volume on SEC approximately corresponded to the molecular weight of a homodimeric species, in line with previous purifications of *Pm*UreE and other UreE homologues (Heimer & Mobley, 2001 ▸; Sriwanthana *et al.*, 1994 ▸). Despite the long, unstructured, histidine-rich C-terminus of full-length UreE, crystals formed in the initial sparse-matrix screens and were optimized to large, rod-like morphologies using micro-seeding (Supplementary Fig. S4). The best crystal diffracted to 1.86 Å resolution, although the data-processing statistics were improved by excluding diffraction data greater than 2.0 Å. The structure was solved using molecular replacement with the *AlphaFold* model of *Pm*UreE (AF-P17090-F1), and two copies of the molecule were found in the asymmetric unit (r.m.s.d. 0.8 Å, 141 C^α^ atoms aligned). The folds of both chains are very similar to the *AlphaFold* model (*A*, r.m.s.d. 1.4 Å, 147 C^α^ atoms aligned; *B*, r.m.s.d. 0.8 Å, 141 C^α^ atoms aligned), with the main differences being in a loop (residues 17–23) and the last four residues in chain *A*. Each of these chains formed the biological dimer through crystallographic symmetry (Fig. 1 ▸). In both chains, continuous density is observed for the folded cores, whereas flexible regions near the termini are not resolved: the C-terminal tails of both chains are absent from the maps, and chain *B* lacks clear density for residues 19–21 near the N-terminus (Supplementary Fig. S5*a*). The final models extend to residue 147 in chain *A* and to residue 144 in chain *B*. Each monomer adopts a two-domain structure connected by a short linker (Fig. 1 ▸*a*). The N-terminal domain (NTD) consists of two short α-helices and two three-stranded mixed β-sheets that stack almost orthogonally to form a rigid core. The C-terminal domain (CTD) comprises an antiparallel four-stranded β-sheet flanked by two α-helices (Fig. 1 ▸*a*). The biological dimer is formed by a head-to-head association of the CTD α3 helices (Pro89–Arg102; Figs. 1 ▸*b* and 1 ▸*c*), with the dimerization stabilized by a combination of hydrophobic and hydrogen-bonding interactions between α3 and β8 (His103–Ala110) of the neighbouring protomer (Fig. 1 ▸*d*). The resolved region of the flexible C-terminus also interacts with α3 of the adjacent protomer (Fig. 1 ▸*d*).

Inspection of the electron-density maps did not reveal convincing density for bound metal ions. No strong, spherical density consistent with a transition metal was observed at the dimer interface or elsewhere, and coordination geometry consistent with Ni(II) was not evident. The crystal structure, therefore, represents an apo form of *Pm*UreE under the conditions used for crystallization. Instead, two irregular, relatively strong density blobs were observed near the N-terminal region of the monomers. Their shapes were elongated/ill-defined rather than spherical. Given the crystallization solution, these features are most consistent with bound buffer components (for example citrate). However, attempts to model them and refine candidate ligands or ions in *Phenix* were not successful. As a result, these sites were left unmodelled in the final coordinates.

### 3.2. *Pm*UreE shares structural similarity with other UreE homologues

**Figure 2:**
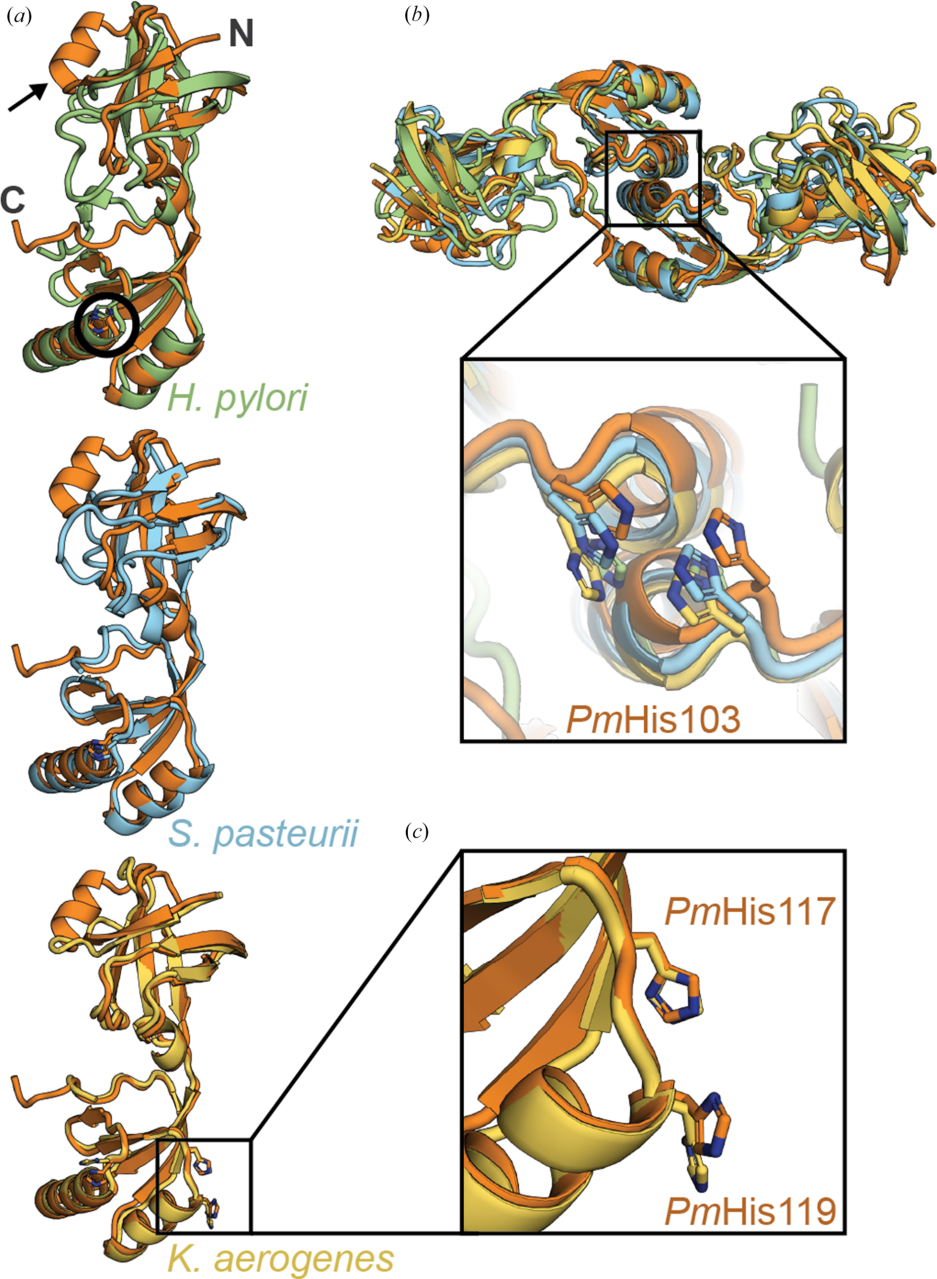
Structural alignment of *Pm*UreE with other UreE homologues. (*a*) Alignment of *H. pylori* UreE (green, PDB entry 3l9z, r.m.s.d. 2.1 Å, 118 C^α^ atoms aligned), *S. pasteurii* UreE (blue, PDB entry 1ear, r.m.s.d. 2.1 Å, 120 C^α^ atoms aligned) and *K. aerogenes* (yellow, PDB entry 1gmu, r.m.s.d. 1.1 Å, 134 C^α^ atoms aligned) against *Pm*UreE (orange). The arrow indicates the additional α-helix in *Pm*UreE and the black circle highlights the position of the conserved histidine residue that sits at the dimer interface. (*b*) Alignment of the dimeric forms of the UreE homologues [coloured as per (*a*)] with the nickel-binding site at the dimerization interface featured (inset). (*c*) An intramolecular secondary nickel-binding site is conserved between *Pm*UreE and *Ka*UreE.

To compare the *Pm*UreE structure with other solved UreE structures, superpositions were generated against *K. aerogenes* UreE (*Ka*UreE; PDB entry 1gmu), *H. pylori* UreE (*Hp*UreE; PDB entry 3l9z) and *S. pasteurii* UreE (*Sp*UreE; PDB entry 1ear) (Fig. 2 ▸*a*). *Pm*UreE shares the greatest structural similarity with *Ka*UreE (r.m.s.d. 1.1 Å, 134 C^α^ atoms aligned), but all four proteins share a conserved two-domain structure and a head-to-head dimer arrangement. Residues in the dimerization interface (*Pm*Pro89–Ala110) are well conserved between *K. aerogenes* and *P. mirabilis* (Supplementary Fig. S1). The overlays indicate that the folded cores align well, with deviations mostly in the N-terminal region and a few solvent-exposed loops (Fig. 2 ▸*a*). Intriguingly, *Pm*UreE has an additional short but well defined α-helix (α1; Fig. 1 ▸*a*, Supplementary Fig. S5*b*) in the N-terminal domain that is a flexible loop in other solved structures. This feature is also present in the *Pm*UreE *AlphaFold* model. Most studies have focused on the metal-binding capabilities of UreE rather than the role of the NTD; however, the NTD is structurally related to heat-shock proteins and has been referred to as a peptide-binding domain (Song *et al.*, 2001 ▸). A recent study showed that the NTD of *Hp*UreE is involved in binding to *Hp*UreG during nickel transfer between the two proteins (Chan *et al.*, 2025 ▸). Although the interaction interface between *Hp*UreE and *Hp*UreG is on the opposite side to α1 in *Pm*UreE, it is possible that this helix could be involved in specialized protein–protein interactions for UreE acquisition or delivery of nickel within *P. mirabilis*.

Given the overall conservation in architecture, we focused our analyses on the two histidine-rich sites that are known to have functional roles in UreE homologues. The first is located at the dimer interface and has been implicated in metal binding across all three homologues. In the *Pm*UreE dimer, *Pm*His103 from each monomer occupies the same interfacial site as the equivalent residues in *Ka*UreE (*Ka*His96), *Hp*UreE (*Hp*His102) and *Sp*UreE (*Sp*His100) (Fig. 2 ▸*b*). The side-chain orientations are closely matched across the four structures, forming a conserved nickel-binding site at the CTD–CTD interface. Mutation experiments have shown that these dimer-interface histidine residues are essential for urease activation in *K. aerogenes* and *H. pylori*, indicating that they are involved in the transfer of nickel to the downstream protein UreG (Colpas *et al.*, 1999 ▸; Colpas & Hausinger, 2000 ▸; Shi *et al.*, 2010 ▸). This was confirmed by the recently released structure of the *Hp*UreE–*Hp*UreG interaction (Chan *et al.*, 2025 ▸).

The second histidine-rich site resides within each C-terminal domain and is associated with metal binding in *K. aerogenes* but is not present in *H. pylori* or *S. pasteurii*. Within each *Pm*UreE monomer, *Pm*His117 and *Pm*His119 align with *Ka*His110 and *Ka*His112 in the CTD (Fig. 2 ▸*c*). The relative positions of the four-stranded β-sheet and adjacent helices are conserved, creating an intramolecular histidine pair analogous to the nickel-binding site reported for *Ka*UreE (Song *et al.*, 2001 ▸). The *Ka*UreE structure was solved with metal bound in these sites (Song *et al.*, 2001 ▸) and mutation of *Ka*His110/*Ka*His112 weakens nickel binding and reduces urease activation *in vivo*, emphasizing the role of the intramolecular site in effective metal trafficking (Colpas *et al.*, 1999 ▸).

### 3.3. Nickel-binding capacity of *Pm*UreE and *Pm*UreE-ΔC17

**Figure 3:**
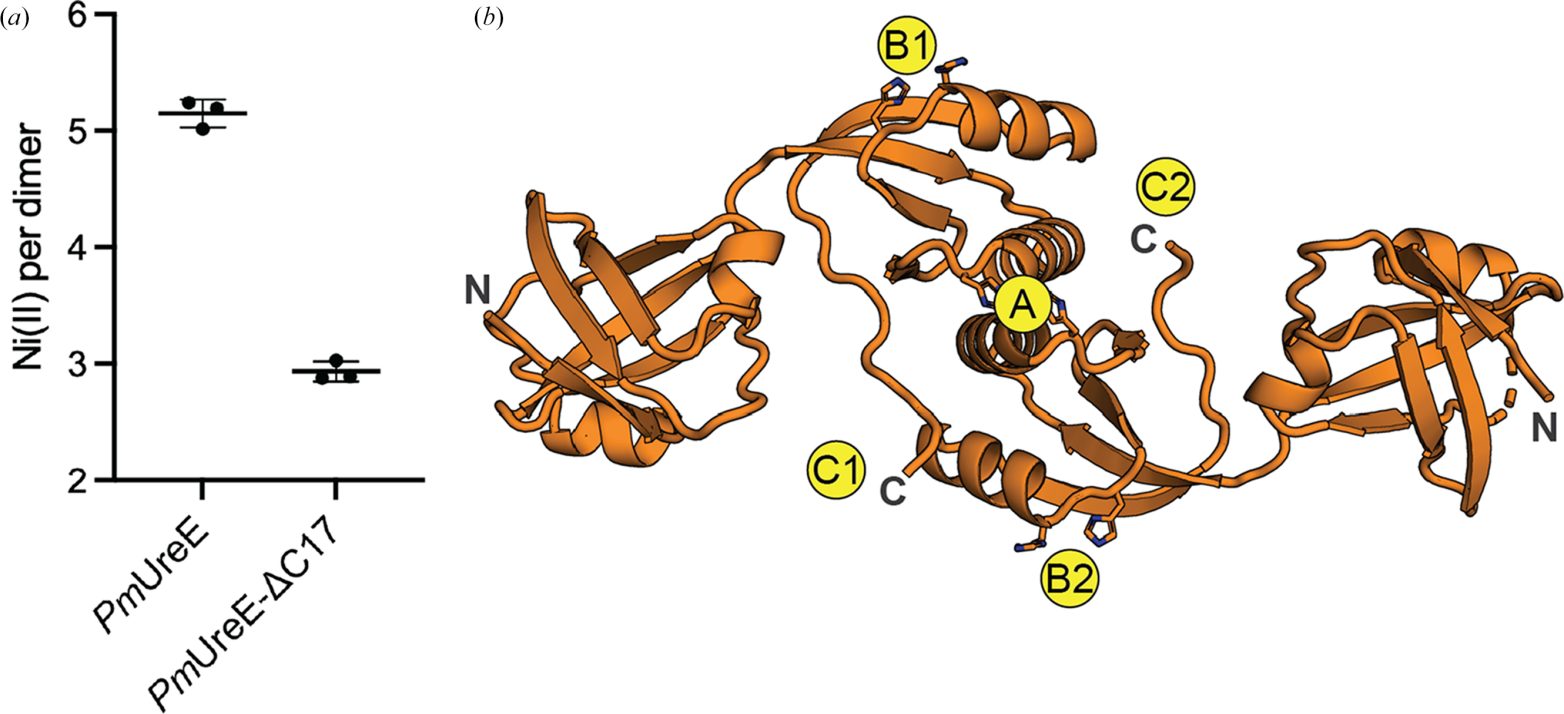
Nickel-binding capacity of *Pm*UreE. (*a*) Quantification of nickel binding to *Pm*UreE and *Pm*UreE-ΔC17 using ICP-MS. The mean and standard deviation of the data points from three replicates are shown for each sample. (*b*) Putative nickel-binding sites in *Pm*UreE based on the ICP-MS results and comparison with previous work on UreE homologues.

Besides the two nickel-binding sites discussed in Section 3.2, the histidine-rich C-terminus, although often unresolved in electron density, has been shown in related systems to increase the total nickel-binding capacity and to modulate the effective stoichiometry (Lee *et al.*, 1993 ▸; Brayman & Hausinger, 1996 ▸). To investigate the nickel-binding stoichiometry of *Pm*UreE and particularly the contribution of the C-terminus, we generated a C-terminally truncated mutant of *Pm*UreE (*Pm*UreE-ΔC17; Supplementary Fig. S3*b*) and analysed the nickel-binding capacity of both wild-type *Pm*UreE and *Pm*UreE-ΔC17. The total nickel content was quantified by ICP-MS after NiCl_2_ incubation and dialysis to remove unbound metal. Across three independent preparations, the Ni(II) content corresponded to 5.15 ± 0.12 Ni(II) ions per *Pm*UreE dimer and 2.93 ± 0.08 Ni(II) ions per *Pm*UreE-ΔC17 dimer under the assay conditions (Fig. 3 ▸*a*). In two of these repeats, the nickel content of the dialysis buffer and 4% HNO_3_ was analysed and found to be BDL or at trace levels (<0.1 µ*M*), so did not contribute to the nickel detected in the samples (Supplementary Table S2). These results suggest that the C-terminus of *Pm*UreE, which contains eight histidine residues (16 per dimer), contributes to the binding of two Ni(II) ions.

The reported stoichiometries by ITC for *Hp*UreE and *Sp*UreE are ∼1 and 2 Ni(II) ions per dimer, respectively (Bellucci *et al.*, 2009 ▸; Zambelli *et al.*, 2013 ▸). In line with their lack of a histidine-rich C-terminal tail and the intramolecular CTD binding site, *Hp*UreE and *Sp*UreE show reduced total nickel binding compared with *Pm*UreE. As expected from sequence and structural alignment, *Pm*UreE [∼5 Ni(II) ions per dimer by ICP-MS] has the most similar nickel-binding capacity to *Ka*UreE, which binds ∼6 Ni(II) ions per dimer based on equilibrium dialysis (Lee *et al.*, 1993 ▸). In *Ka*UreE, the flexible C-terminus has ten histidine residues (contributing 20 per dimer), compared with the eight in *Pm*UreE. A C-terminally truncated form of *Ka*UreE (H144*) binds only ∼2 Ni(II) ions per dimer, which also aligns with our measured data for the *Pm*UreE-ΔC17 mutant [∼3 Ni(II) ions per dimer] (Lee *et al.*, 1993 ▸; Brayman & Hausinger, 1996 ▸). These results suggest that an extra two histidine residues in each flexible C-terminus allow the binding of two more Ni(II) ions per dimer. The C-terminal histidine residues are thought to be involved in maintaining an increased local concentration of Ni(II) ions for transfer to urease. Although the loss of this region results in reduced stoichiometry, removal of the flexible C-terminus has been shown to not affect urease activation in *K. aerogenes* (Mulrooney *et al.*, 2005 ▸; Brayman & Hausinger, 1996 ▸) or the transfer of nickel onto UreG in *H. pylori* (Chan *et al.*, 2025 ▸). However, the addition of a histidine-rich C-terminal segment onto *Hp*UreE does increase the urease activity and recent work has indicated that the flexible C-terminus is required for the UreE acquisition of nickel from HypA in *H. pylori* (Chan *et al.*, 2025 ▸). Whether the C-terminal region has a similar role in other species is yet to be determined.

### 3.4. Putative *Pm*UreE nickel-binding sites

The structural alignment of UreE homologues (Fig. 2 ▸), along with the measured stoichiometries, supports the assignment of putative nickel-binding sites in *Pm*UreE (Fig. 3 ▸*b*). Site A is assigned to the dimer-interface pocket, equivalent to the interface site identified across all UreE structures for metal capture (Remaut *et al.*, 2001 ▸; Song *et al.*, 2001 ▸; Shi *et al.*, 2010 ▸; Banaszak *et al.*, 2012 ▸; Zambelli *et al.*, 2013 ▸; Fig. 3 ▸*b*). Sites B1 and B2 correspond to intramolecular sites within each monomer, positioned by the conserved histidine pair that aligns with *Ka*UreE (Fig. 2 ▸*c*). Together, the A/B sites account for three core nickel-binding positions per dimer. The remaining capacity can be explained by the histidine-rich C-terminus: C1 and C2 represent the potential C-terminal-associated nickel-binding sites, consistent with the high total nickel per dimer in the wild type and the loss of ∼2 Ni(II) ions after C-terminal truncation, as observed during ICP-MS (Fig. 3 ▸*a*). As there was no corresponding electron density detected for the flexible C-terminus, the precise histidine residues contributing to the C1/C2 sites cannot be assigned. With the eight histidine residues in the *Pm*UreE C-terminal region (Supplementary Fig. S1), there are several alternative sites or binding modes that could operate, and further experimentation will be required to clarify this.

## 4. Conclusion

Overall, this study has solved the structure of *Pm*UreE at 2.0 Å resolution, identifying a conserved *Pm*UreE dimer scaffold with three conserved sites that likely function in metal binding and transfer. ICP-MS data support multi-site nickel binding with a significant contribution from the histidine-rich C-terminal tail, with the ΔC17 truncation decreasing the total nickel-binding capacity. Together, these data validate *Pm*UreE as a metallochaperone that captures Ni(II) ions. We also provide a foundation for understanding the role of UreE in *P. mirabilis* pathogenesis and evaluating its potential as a target for new antibacterial therapies in CAUTIs.

## Supplementary Material

PDB reference: *Proteus mirabilis* UreE, 9zlo

Supplementary Tables and Figures. DOI: 10.1107/S2059798326001907/ag5064sup1.pdf

Raw X-ray diffraction data for PDB entry 9lzo.: https://doi.org/10.5281/zenodo.18652652

## Figures and Tables

**Table 1:** Crystallographic statistics for the *Pm*UreE structure Values in parentheses are for the highest resolution shell.

PDB code	9zlo
Data collection	
Diffraction source	MX2, Australian Synchrotron
Wavelength (Å)	0.9537
Temperature (K)	100
Detector	Dectris EIGER 16M
Crystal-to-detector distance (mm)	250
Total rotation range (°)	360
Exposure time per degree (s)	0.1
Exposure time per image (s)	0.01
Space group	*P*2_1_2_1_2_1_
*a*, *b*, *c* (Å)	38.39, 89.98, 106.97
α, β, γ (°)	90, 90, 90
Mosaicity (°)	0.15
Resolution range (Å)	45.98–2.00 (2.05–2.00)
Total No. of reflections	341566 (23467)
No. of unique reflections	25897 (1868)
Completeness (%)	100.0 (100.0)
Multiplicity	13.2 (12.6)
〈*I*/σ(*I*)〉 from merged data	15.5 (3.3)
CC_1/2_	0.999 (0.903)
*R*_meas_	0.111 (0.848)
Overall *B* factor from Wilson plot (Å^2^)	28.48
Refinement	
Resolution range (Å)	45.98–2.00 (2.08–2.00)
Completeness (%)	99.97 (100.0)
No. of reflections, working set	25832 (2798)
No. of reflections, test set	1314 (141)
Final *R*_work_	0.1871 (0.2117)
Final *R*_free_	0.2200 (0.2544)
No. of non-H atoms	
Protein	2244
Waters	194
Total	2438
R.m.s. deviations from ideality	
Bond lengths (Å)	0.007
Angles (°)	0.87
Average *B* factors (Å^2^)	
Protein	35.48
Waters	37.55
Ramachandran plot	
Favoured regions (%)	98.94
Outliers (%)	0.0
Clashscore	2.88

## Data Availability

The *Pm*UreE crystallographic model and structure factors have been deposited in the Protein Data Bank with the accession code 9zlo. The raw X-ray diffraction data can be accessed through Zenodo (https://doi.org/10.5281/zenodo.18652652.
