# Supplementary material for: Molecular structure and nickel-binding capacity of Proteus mirabilis UreE
Source: Acta Crystallogr D Struct Biol. 2026 Mar 16;82(Pt 4):348–57. doi: 10.1107/S2059798326001907 (PMC13044922; doi:10.1107/S2059798326001907)
Supplement: Supplementary file 1 [file d-82-00348-sup1.pdf]

STRUCTURAL BIOLOGY

Volume 82 (2026)

Supporting information for article:

Molecular structure and nickel-binding capacity of *Proteus mirabilis* UreE

Jiayi Pan, Sarah L. Mueller, Nuren Tasneem, Yang Wu and Emily J. Furlong

Table S1 Primers used for cloning

Sequences are shown 5’→3’. “Insertion” primers amplify the gene insert; “Backbone” primers amplify the vector. The ΔC17 reverse primer encodes a stop codon at the truncation site to remove the last 17 amino acid residues. “Forward/Reverse” indicate primer orientation relative to the coding sequence.

Construct	Primer name	Sequence (5’→3’)
**pETHis6TEVLIC_UreE**	Insertion (*ureE* ORF) Forward	CTTTAAGAAGGAGATATACCATGAAAAAATTTACTCAG ATTATTGATCAACAAAAAG
	Insertion (*ureE* ORF) Reverse	CTCAGTGGTGGTGGTGGTGGTGTTAATGGTGGTGAT CATGGTGGTG
	Backbone Forward	CACCACCACCACCACCAC
	Backbone Reverse	CATGGTATATCTCCTTCTTAAAGTTAAAC
**pETHis6TEVLIC_UreE-ΔC17**	Forward	GCTTATGGTGGATCATCCGG
	Reverse	TTACCCCGGCTCAGGTTG
**Colony PCR**	T7 promoter	GCGAAATTAATACGACTCACTATAGG
	T7 terminator	CCCCTCAAGACCCGTTTAG

Table S2 ICP-MS data analysis.

***Pm*UreE**

Sample	Dimeric Protein Conc. (µM)			Measured Ni(II) (µM)			Ni(II) normalized to 5 µM protein (µM)			Ni(II) per dimer		
	R1	R2	R3	R1	R2	R3	R1	R2	R3	R1	R2	R3
Ni(II)-incubated protein	5.49	4.36	5.94	27.53	22.63	31.14	25.07	25.95	26.2	5.014	5.19	5.24
Dialysis buffer		/		0.0658	NM**	0.0045		/			/	
4 % HNO3		/		BDL*	NM**	BDL*		/			/	

PmUreE-ΔC17

Sample	Dimeric Protein Conc. (µM)			Measured Ni(II) (µM)			Ni(II) normalized to 5 µM protein (µM)			Ni(II) per dimer		
	R1	R2	R3	R1	R2	R3	R1	R2	R3	R1	R2	R3
Ni(II)-incubated protein	4.47	4.18	4.77	12.86	12.65	13.78	14.38	15.13	14.43	2.88	3.03	2.89
Dialysis buffer		/		0.0022	NM**	0.0045		/			/	
4 % HNO3		/		0.0038	NM**	BDL*		/			/	

*Below Detection Limit

** Not Measured

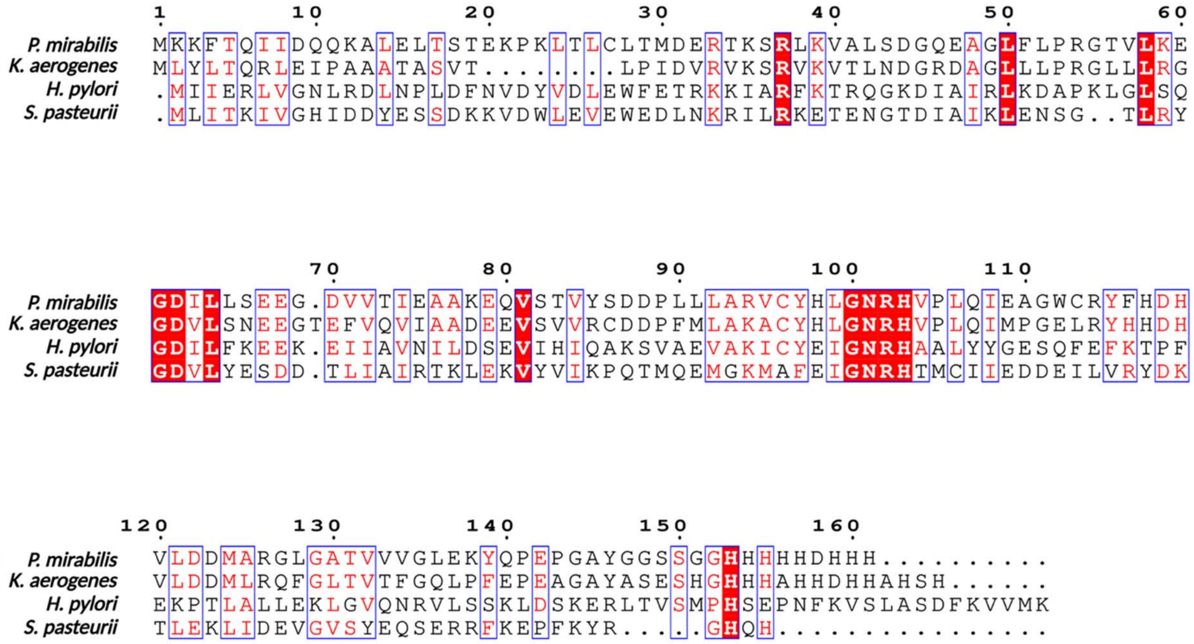

Figure S1 Sequence alignment of UreE homologues. Multiple-sequence alignment of UreE from *P. mirabilis*, *K. aerogenes*, *H. pylori*, and *S. pasteurii*. Residues identical in all four species are highlighted with a red background; residues conserved in three species are outlined with a blue box. Residue numbers follow the *P. mirabilis* sequence.

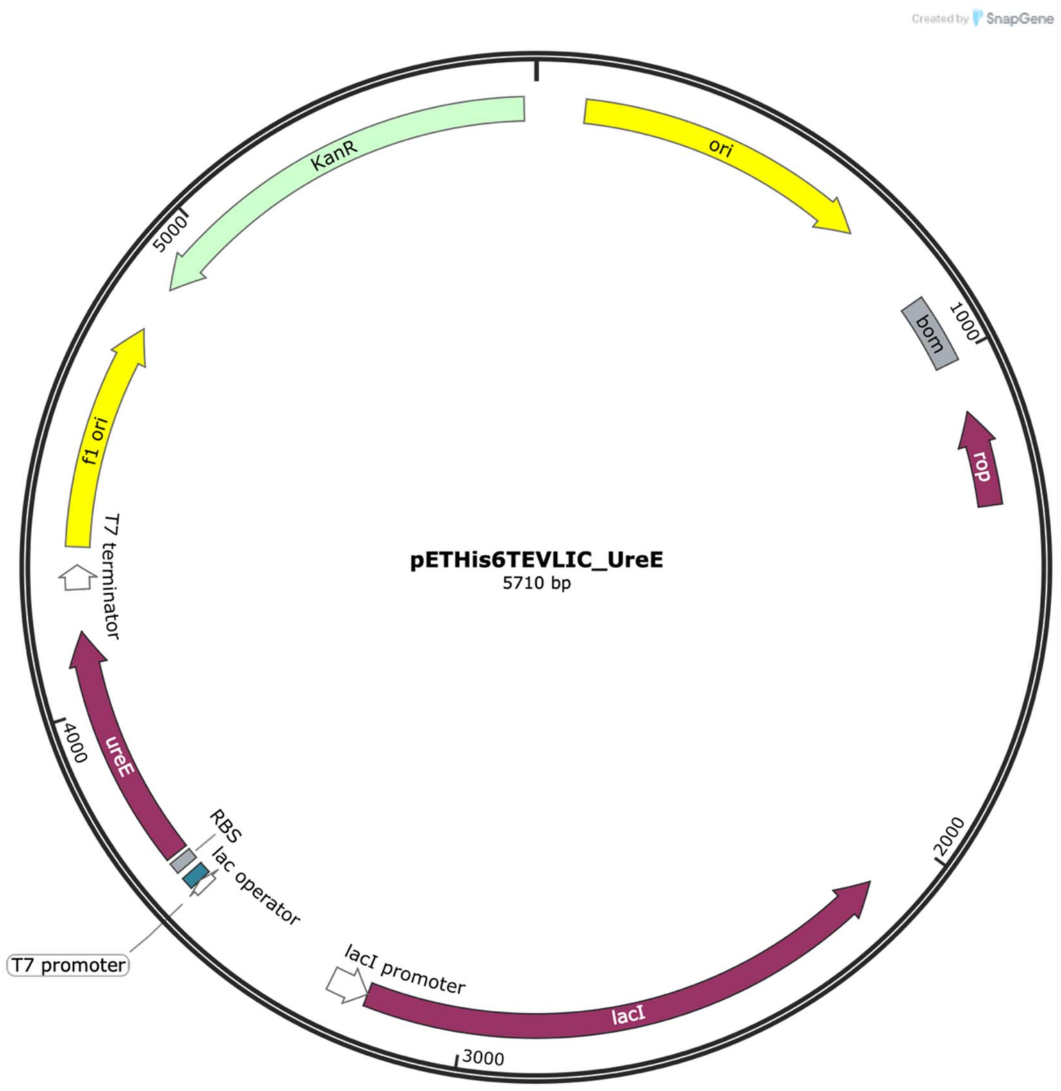

Figure S2 Map of *Pm*UreE expression plasmid. The *Pm*UreE-ΔC17 expression plasmid was identical except for the addition of a stop codon in the DNA after Gly144 in the C-terminal region. Figure created in SnapGene.

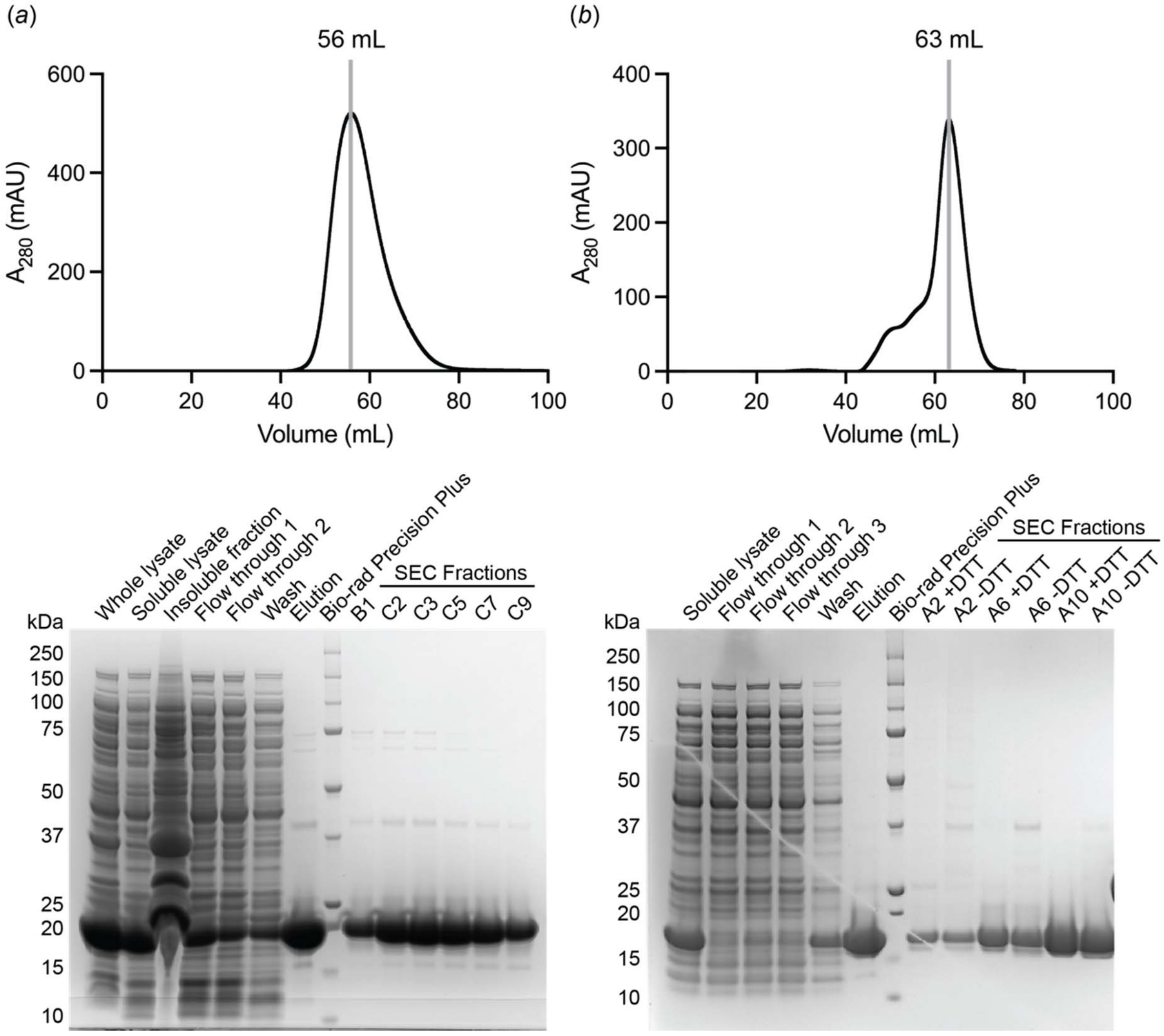

Figure S3 Purification of *Pm*UreE and *Pm*UreE-ΔC17**.** (*a*) Size-exclusion chromatography (SEC) profile of wild-type *Pm*UreE on a HiLoad 16/600 Superdex 75 column; UV absorbance was monitored at 280 nm. The vertical line marks the elution peak of the protein. Below, SDS-PAGE of all purification steps. SEC fractions showed a single dominant band at ~18 kDa, corresponding to the expected molecular weight. (*b*) SEC profile of the C-terminal truncation variant *Pm*UreE-ΔC17 on a new HiLoad 16/600 Superdex 75 column with the SDS-PAGE of all purification steps below. SDS-PAGE of the SEC fractions showing a single dominant band at ~16 kDa, corresponding to the expected monomer molecular weight. The larger than expected difference in elution volume between the proteins is likely due to a new column being used for (*b*), which resulted in better resolution of the peaks.

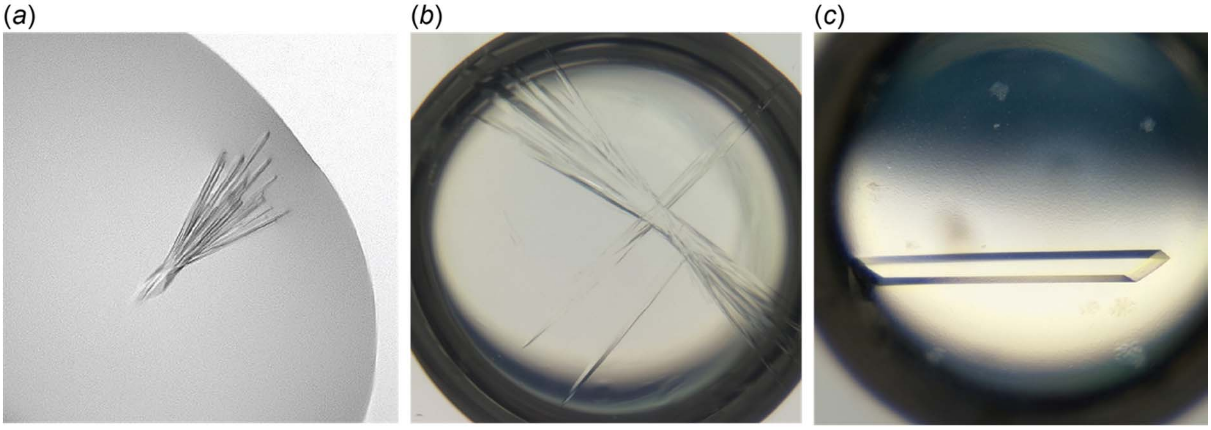

Figure S4 Progression of *Pm*UreE crystallisation from initial hit to seeded optimisation. (*a*) The initial sparse-matrix screen yielded a single crystallisation hit, resulting in thin needle crystals (Condition: 0.2 M ammonium citrate dibasic; 20% w/v polyethylene glycol (PEG) 3350). The image was recorded from a sitting-drop vapour-diffusion trial. (*b*) First-round optimisation around the initial condition generated longer needles (Condition: 0.14 M ammonium citrate dibasic, 18% w/v PEG 3350, with 100 µM $NiCl_2$ added; a representative well is shown). The image was recorded from hanging-drop vapour-diffusion trials. (*c*) Microseeding using crushed microcrystals from the first-round optimisation markedly improved crystal quality, producing thicker, well-formed single crystals (Condition: 0.1 M sodium citrate, pH 5.0, 19% w/v PEG 6000). The image was recorded from hanging-drop vapour-diffusion trials.

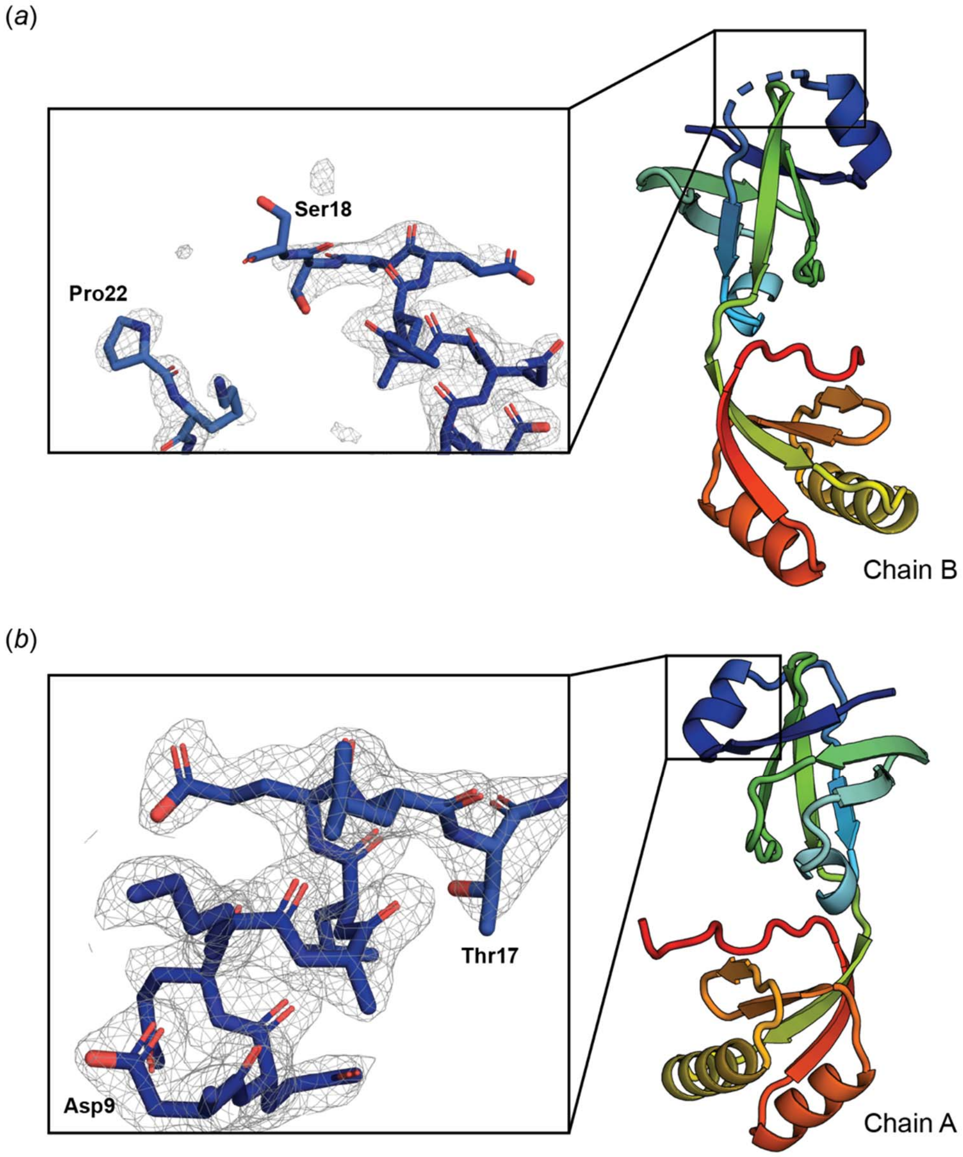

Figure S5 Key areas of electron density in the *Pm*UreE crystal structure. (*a*) The 2*m*F_o-*D*F_c electron density map around the missing residues 19-21 in Chain B, contoured at 1.0 σ. (*b*) The 2*m*F_o-*D*F_c electron density map for the α1 helix in Chain A, contoured at 1.0 σ, carve = 2.0. The maps were generated using phenix.mtz2map.
